# Gene therapy restores adipose tissue and metabolic health in a pre-clinical mouse model of lipodystrophy

**DOI:** 10.1016/j.omtm.2022.09.014

**Published:** 2022-10-03

**Authors:** Nadine Sommer, Ahlima Roumane, Weiping Han, Mirela Delibegović, Justin J. Rochford, George D. Mcilroy

**Affiliations:** 1 The Rowett Institute, University of Aberdeen, Aberdeen AB25 2ZD, UK; 2 Aberdeen Cardiovascular and Diabetes Centre, University of Aberdeen, Aberdeen AB25 2ZD, UK; 3 School of Pharmacy and Biomedical Sciences, Curtin Health Innovation Research Institute, Curtin University, Perth, WA 6102, Australia; 4 Institute of Molecular and Cell Biology, Agency for Science, Technology and Research (A∗STAR), 138667 Singapore, Singapore; 5 Center for Neuro-Metabolism and Regeneration Research, Guangzhou Regenerative Medicine and Health Guangdong Laboratory, Guangzhou 510700, China; 6 School of Laboratory Medicine and Life Sciences, Wenzhou Medical University, Wenzhou, Zhejiang 325035, China; 7 Institute of Medical Sciences, University of Aberdeen, Aberdeen AB25 2ZD, UK

**Keywords:** adeno-associated virus, adipose tissue, BSCL2, gene therapy, lipodystrophy, metabolic disease, type 2 diabetes

## Abstract

Congenital generalized lipodystrophy type 2 is a serious multisystem disorder with limited treatment options. It is caused by mutations affecting the *BSCL2* gene, which encodes the protein seipin. Patients with congenital generalized lipodystrophy type 2 lack both metabolic and mechanical adipose tissue and develop severe metabolic complications including hepatic steatosis, lipoatrophic diabetes, and cardiovascular disease. Gene therapies are becoming viable treatments, helping to alleviate inherited and acquired human disorders. We aimed to determine whether gene therapy could offer an effective form of medical intervention for lipodystrophy. We examined whether systemic adeno-associated virus delivery of human *BSCL2* could reverse metabolic disease in seipin knockout mice, where white adipose tissue is absent. We reveal that adeno-associated virus gene therapy targets adipose progenitor cells *in vivo* and substantially restores white adipose tissue development in adult seipin knockout mice. This resulted in both rapid and prolonged beneficial effects to metabolic health in this pre-clinical mouse model of congenital generalized lipodystrophy type 2. Hyperglycemia was normalized within 2 weeks post-treatment together with normalization of severe insulin resistance. We propose that gene therapy offers great potential as a therapeutic strategy to correct multiple metabolic complications in patients with congenital lipodystrophy.

## Graphical abstract

**Figure:**
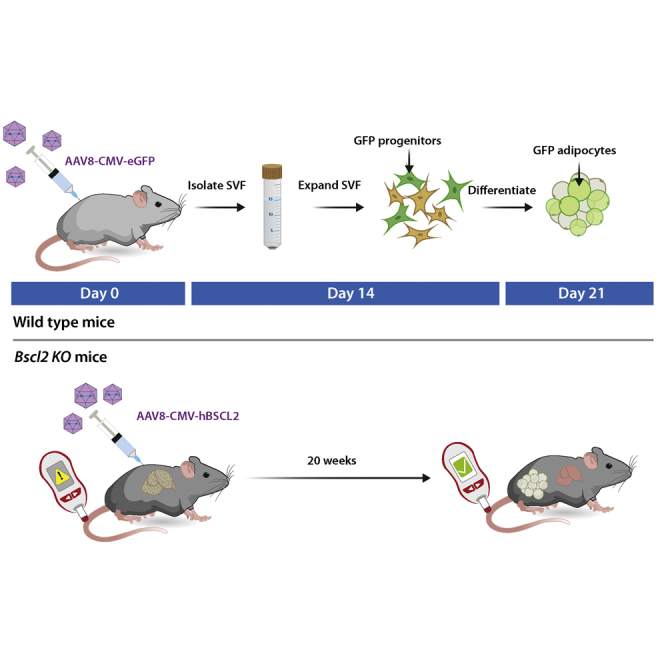

## Teaser

Lipodystrophy is a rare disorder of adipose tissue deficiency with severe metabolic complications. We demonstrate that AAV-mediated gene therapy restores adipose tissue development and improves metabolic health in a pre-clinical mouse model of congenital generalized lipodystrophy. Gene therapy offers great potential as a therapeutic strategy for patients suffering from lipodystrophy.

## Introduction

Gene therapy offers the opportunity to provide a targeted long-lasting treatment for multiple human disorders and diseases. Importantly, the number of gene therapeutic strategies gaining approved drug status in Europe and the United States continues to grow.^1^ Lipodystrophies are a group of genetic or acquired conditions affecting the development and function of adipose tissue. Due to the critical role played by adipose tissue in the maintenance of metabolic homeostasis,^2^ patients with lipodystrophy develop severe metabolic complications. These include hepatic steatosis, type 2 diabetes, and cardiovascular disease as well as a range of additional co-morbidities such as increased appetite, chronic pain, and fatigue.^3^ Current therapies used to manage lipodystrophy do not alleviate all aspects of the condition, and the most effective treatment, metreleptin, requires daily injections and is not widely available. Congenital generalized lipodystrophy (CGL) is a rare disorder, and the precise worldwide prevalence is unknown. However, estimations have been generated using case reports, literature searches, and electronic medical record database searches, which indicate a prevalence of approximately 0.1–1 cases/million for CGL.^4^, ^5^, ^6^ The most severe form of lipodystrophy is CGL type 2 (CGL2), which is caused by mutations affecting *BSCL2*/seipin.^7^ Multiple groups have generated seipin knockout (SKO) mouse models,^8^, ^9^, ^10^, ^11^ which broadly recapitulate the metabolic phenotype observed in patients with CGL2. Previous studies have revealed that the restoration of adipose tissue is sufficient to reverse metabolic disease in SKO mice. This has been achieved through genetic modification or adipose transplantation.^12^, ^13^, ^14^ Other groups have effectively employed adeno-associated virus (AAV)-mediated gene therapeutic strategies that target developed adipose tissue depots in order to alleviate metabolic disease caused by obesity.^15^, ^16^, ^17^ Therefore, we hypothesized that gene therapy could offer a novel, safe, and effective treatment to reverse severe metabolic complications that develop in patients with lipodystrophy. We investigated whether systemic AAV-mediated delivery of human *BSCL2*/seipin could restore adipose tissue development and improve metabolic health in a pre-clinical mouse model of CGL2.

## Results

### AAV delivery to murine tissues

**Figure 1:**
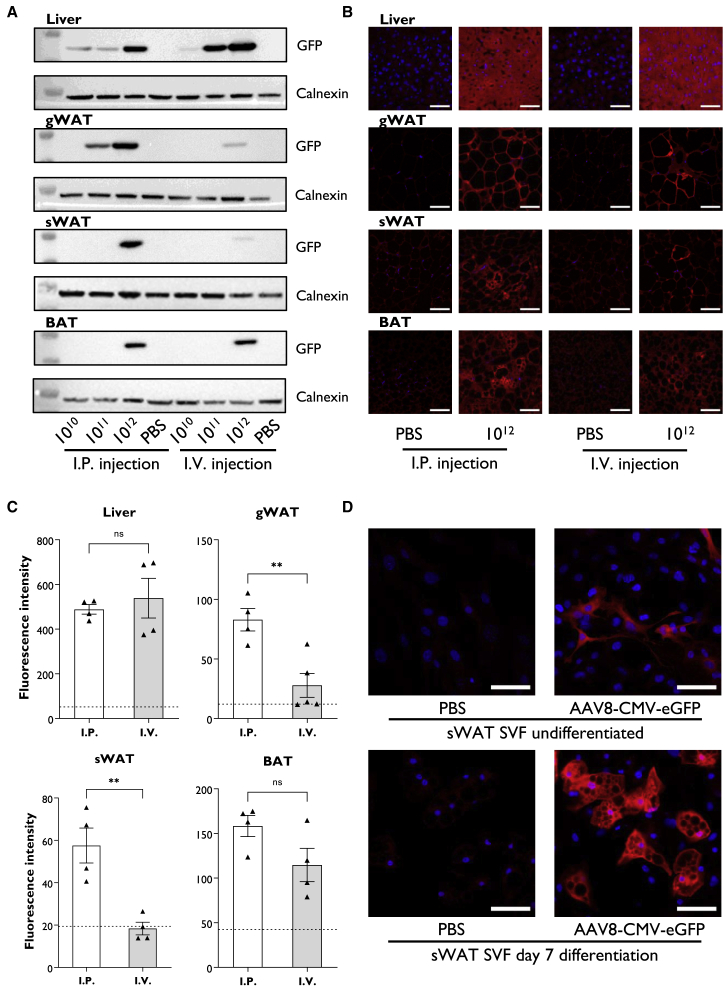
Adeno-associated virus delivery of eGFP to murine tissues (A) Western blot analysis of EGFP in liver, gonadal white adipose tissue (gWAT), subcutaneous WAT (sWAT), and brown adipose tissue (BAT). Male mice were injected with AAV8 vectors expressing EGFP from the mammalian cytomegalovirus (CMV) promoter (AAV8-CMV-EGFP). Intraperitoneal (i.p.) or intravenous (i.v.) injections were given using 1 × 10^10^, 1 × 10^11^, or 1 × 10^12^ genome copies of AAV, and an equivalent volume of PBS was used as a control. (B) Representative immunohistochemistry sections of EGFP in liver, gWAT, sWAT, and BAT in mice receiving 1 × 10^12^ genome copies of AAV8-CMV-EGFP or an equivalent volume of PBS by i.p. or i.v. Scale bar represents 50 μm. (C) Quantification of EGFP fluorescence intensity from samples presented in (B). Dashed line represents background fluorescence detected in PBS samples of corresponding tissues. Data presented as the mean ± SEM, n = 4–5 different fields of view per condition, ∗∗p < 0.01 i.p. versus i.v. (D) Immunofluorescence of EGFP in stromal vascular fraction (SVF) primary cell cultures isolated from sWAT of mice i.p. injected with 1 × 10^12^ genome copies of AAV8-CMV-EGFP or PBS. SVF cultures were grown to confluency (undifferentiated) and induced to differentiate to adipocytes for 7 days. Scale bar represents 50 μm.

Ten-week-old male C57BL/6J mice were intraperitoneally (i.p.) or intravenously (i.v.) injected with 1 × 10^10^, 1 × 10^11^, or 1 × 10^12^ genome copies of AAV8-CMV-EGFP or an equivalent volume of PBS. Tissues were collected 2 weeks later. Western blot analysis revealed EGFP expression at all doses in the livers of i.p. and i.v. injected mice. At 1 × 10^12^, EGFP was detectable in gonadal white adipose tissue (gWAT), subcutaneous WAT (sWAT), and brown adipose tissue (BAT) by i.p. and i.v. injection. i.p. administration produced stronger expression within WAT depots, and EGFP was observed in gWAT at 1 × 10^11^ by i.p. (Figure 1A). EGFP expression was detectable in the heart and testis at a dose of 1 × 10^12^ by i.p. or i.v., while little or no expression was observed in the muscle or kidney (Figure S1A). Effective targeting of adipose tissues was confirmed by immunohistochemistry (Figure 1B), and quantification revealed that i.p. injection produced significantly greater EGFP expression in gWAT and sWAT compared with i.v. injection (Figure 1C).

Our findings confirm multiple reports that AAV vectors can effectively target adipose tissue.^18^ However, it is unclear if adipose progenitor cells are also targeted by this method *in vivo*. We therefore i.p. injected 10-week-old C57BL/6J male mice with 1 × 10^12^ genome copies of AAV8-CMV-EGFP or PBS and isolated the stromal vascular fraction (SVF) from gWAT and sWAT 2 weeks later. Expression of EGFP was evident in gWAT and sWAT primary cultures (Figure S1B). We expanded sWAT primary cells and induced adipocyte differentiation. Expression of EGFP remained detectable in confluent undifferentiated sWAT SVF cultures despite extensive proliferation. Similarly, EGFP-positive adipocytes containing lipid droplets were evident at day 7 of differentiation (Figure 1D).

We therefore hypothesized that gene therapy may be an effective strategy to treat a pre-clinical mouse model of CGL2, where adipogenesis is predicted to have stalled.^19^^,^^20^ As this has not previously been investigated, we combined the strong cytomegalovirus (CMV) promoter with an AAV8 serotype vector, which has been shown to effectively target adipose tissue.^18^ This was used to overexpress the long form of the human *BSCL2* gene (AAV8-CMV-hBSCL2; Figure S1C) and determine if AAV-mediated gene therapy could restore adipose tissue development and improve metabolic health in a mouse model of CGL2.

### Gene therapy prevents weight gain and hyperglycemia

**Figure 2:**
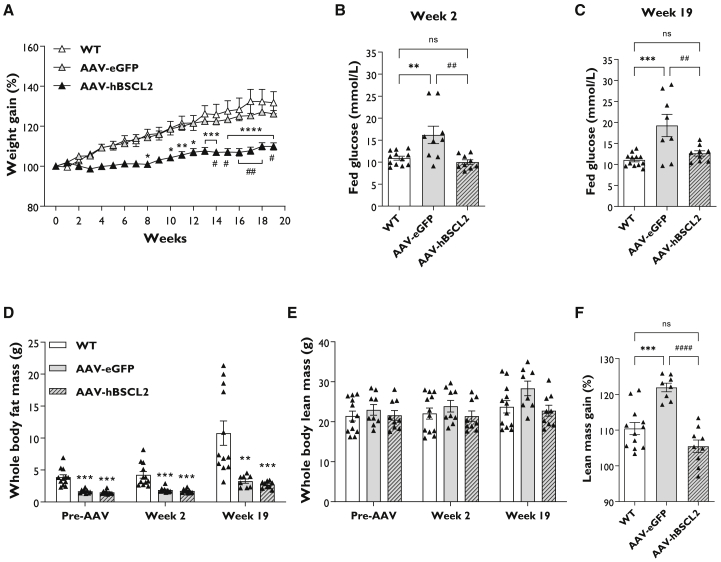
Gene therapy prevents weight gain and rescues hyperglycemia in seipin knockout mice (A) Weight gain progression in wild-type (WT) and seipin knockout (SKO) mice after i.p. injection of 1 × 10^12^ genome copies of AAV8 vectors containing EGFP (AAV-eGFP) or hBSCL2 (AAV-hBSCL2). (B and C) Serum glucose levels in *ad lib* fed WT, AAV-EGFP, and AAV-hBSCL2 mice at 2 (B) and 19 (C) weeks after administration of gene therapy. (D and E) Whole-body fat mass (D) and whole-body lean mass (E) levels assessed by Echo-MRI prior to gene therapy (pre-AAV) 2 and 19 weeks after AAV administration. (F) Percentage of lean mass gained 19 weeks after AAV administration compared with pre-AAV lean mass values. All data are biological replicates presented as the mean ± SEM, n = 12 (WT), 8–9 (AAV-EGFP), and 9 (AAV-hBSCL2) mice per group, ∗p < 0.05, ∗∗p < 0.01, ∗∗∗p < 0.001, and ∗∗∗∗p < 0.0001 versus WT, #p < 0.05, ##p < 0.01, and ####p < 0.0001 versus AAV-EGFP.

Cohorts of 10- to 14-week-old male and female SKO mice were randomized and i.p. injected with 1 × 10^12^ genome copies of AAV8-CMV-hBSCL2 (AAV-hBSCL2) or AAV8-CMV-EGFP (AAV-EGFP). Mice were fed a chow diet for 20 weeks while physiological and metabolic measurements were performed (Figure S1D). Weight gain in wild-type (WT) and AAV-EGFP mice increased 25%–30% compared with levels prior to AAV administration, and no significant differences were observed. This has been observed previously^8^, ^9^, ^10^, ^11^ and is likely attributable to increased hepatomegaly/lean mass gain observed in SKO mice. In contrast, AAV-hBSCL2 mice gained less weight, which became apparent 3 weeks post-treatment, with significant differences observed in AAV-hBSCL2 compared with WT and AAV-EGFP from 8 and 14 weeks, respectively (Figure 2A). Examination of blood glucose levels in *ad lib* fed mice revealed that AAV-EGFP mice were hyperglycemic; this was significantly rescued by AAV-hBSCL2 at 2- and 19-weeks post-injection. Glucose levels in AAV-hBSCL2 were not significantly different compared with WT mice at either time point (Figures 2B and 2C).

As expected, Echo-MRI analysis revealed that SKO mice had significantly decreased whole-body fat mass compared with WT controls prior to AAV administration (Figure 2D). This remained significantly decreased at 2 and 19 weeks after administration of AAV. No significant differences were detectable between AAV-EGFP and AAV-hBSCL2 (Figure 2D). Similar results were observed when whole-body fat levels were normalized to body weight (Figure S2A). Whole-body lean mass levels normalized to body weight were significantly increased in SKO mice compared with WT controls. No significant differences were found between AAV-EGFP and AAV-hBSCL2 (Figure S2B). Increased absolute whole-body lean mass was apparent in AAV-EGFP but not AAV-hBSCL2 at week 19; however, this was not significant (Figure 2E). When the percentage of lean mass gained for each animal was calculated, AAV-EGFP mice gained significantly more lean mass compared with WT controls over the 19-week period. AAV-hBSCL2 mice, however, failed to similarly increase lean mass levels, which were significantly lower than AAV-EGFP (Figure 2F).

### Gene therapy prevents hepatomegaly and restores WAT development

**Figure 3:**
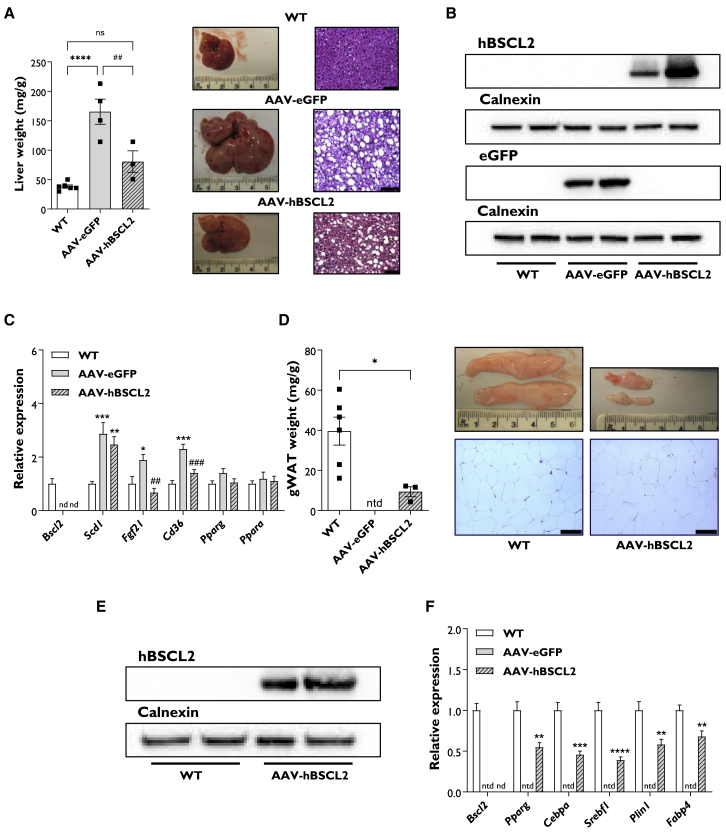
Gene therapy prevents hepatomegaly and restores WAT development in SKO mice (A and D) Tissue weight, dissection images, and representative H&E sections of liver (A) and gWAT (D) from WT, AAV-EGFP, and AAV-hBSCL2 male mice. Scale bar represents 80 μm. (B and E) Western blot analysis of EGFP and hBSCL2 protein levels in liver (B) and hBSCL2 protein levels in gWAT (E) in WT, AAV-EGFP, and AAV-hBSCL2 mice. One male and one female are presented in each condition. (C and F) Relative gene expression levels of metabolic markers in the liver (C) and markers of adipogenesis in gWAT (F) from WT, AAV-EGFP, and AAV-hBSCL2 male and female mice. All data are from 20 weeks after AAV administration and are biological replicates presented as the mean ± SEM, n = 6 (WT), 4 (AAV-EGFP), and 3 (AAV-hBSCL2) mice per group for (A) and (D), n = 11 (WT), 8 (AAV-EGFP), and 9 (AAV-hBSCL2) for (C) and (F), ∗p < 0.05, ∗∗p < 0.01, ∗∗∗p < 0.001, and ∗∗∗∗p < 0.0001 versus WT, ##p < 0.01 and ###p < 0.001 versus AAV-EGFP. ntd, no tissue dissected; nd, not detected.

AAV-EGFP mice had significantly increased liver weights compared with WT controls, as expected given the substantial hepatic steatosis known to occur in this model of CGL2, mimicking the human condition.^8^, ^9^, ^10^, ^11^ Liver weights in AAV-hBSCL2 mice were significantly decreased compared with AAV-EGFP 19 weeks after AAV-hBSCL2 injection. H&E staining also appeared to indicate lower lipid accumulation within the livers of AAV-hBSCL2 compared with AAV-eGFP. Nonetheless, lipid droplets were still readily apparent in AAV-hBSCL2 livers (Figure 3A). Western blot analysis revealed that EGFP and hBSCL2 protein was detectable in the liver 5 months post-AAV injection (Figure 3B). Murine BSCL2 protein was not detected in WT mice due to use of a human-specific BSCL2/seipin antibody. Liver mRNA transcript levels of EGFP and *hBSCL2* were also evident (Figure S2C), while murine *Bscl2* mRNA was confirmed to be absent in SKO mice (Figure 3C). We next examined hepatic markers known to be dysregulated in SKO mice.^21^^,^^22^ The *de novo* lipogenesis marker *Scd1* was significantly upregulated in SKO mice compared with WT controls. Injection of SKO mice with AAV-hBSCL2 had no effect on expression levels of this marker (Figure 3C). We found no significant differences when examining *Pparg* or *Ppara* expression. Expression of *Fgf21* and the fatty acid transporter *Cd36* was significantly increased in AAV-EGFP mice compared with WT controls. Both markers were normalized in AAV-hBSCL2 mice, with expression significantly decreased compared with AAV-eGFP (Figure 3C).

Despite no significant alterations in whole-body lipid content between AAV-EGFP and AAV-hBSCL2 (Figure 2D), post-mortem examination revealed that AAV-hBSCL2 injection led to substantial visceral WAT development. Adipose tissues from gWAT (Figure 3D) and retroperitoneal WAT (rWAT) depots (Figure S2D) in AAV-hBSCL2 mice were present, which were completely absent in AAV-EGFP mice. H&E sections indicated that both gWAT and rWAT adipocyte size and morphology were like that observed in WT mice (Figures 3D and S2D). Small amounts of BAT were detectable in SKO mice, which were significantly reduced compared with WT mice and not significantly changed by AAV-hBSCL2 injection. H&E staining indicated that large unilocular lipid droplets were present in both WT and AAV-hBSCL2 mice, reminiscent of BAT morphology under thermoneutral conditions. Triglyceride storage was dramatically increased in AAV-hBSCL2 mice compared with AAV-EGFP controls (Figure S2E). Western blot analysis revealed that human seipin protein was detectable in rescued gWAT of AAV-hBSCL2-injected mice (Figure 3E). This was verified by qPCR analysis of h*BSCL2* transcript levels (Figure S2F). We confirmed the absence of murine *Bscl2* expression and examined the expression of key transcription factors (*Pparg*, *Cebpa*, and *Srebf1*) and markers of adipogenesis (*Plin1* and *Fabp4*) in rescued gWAT of AAV-hBSCL2 mice. Substantial expression was evident for all markers examined; however, levels were significantly lower than those detected in WT mice (Figure 3F).

### Insulin sensitivity is restored in SKO mice

**Figure 4:**
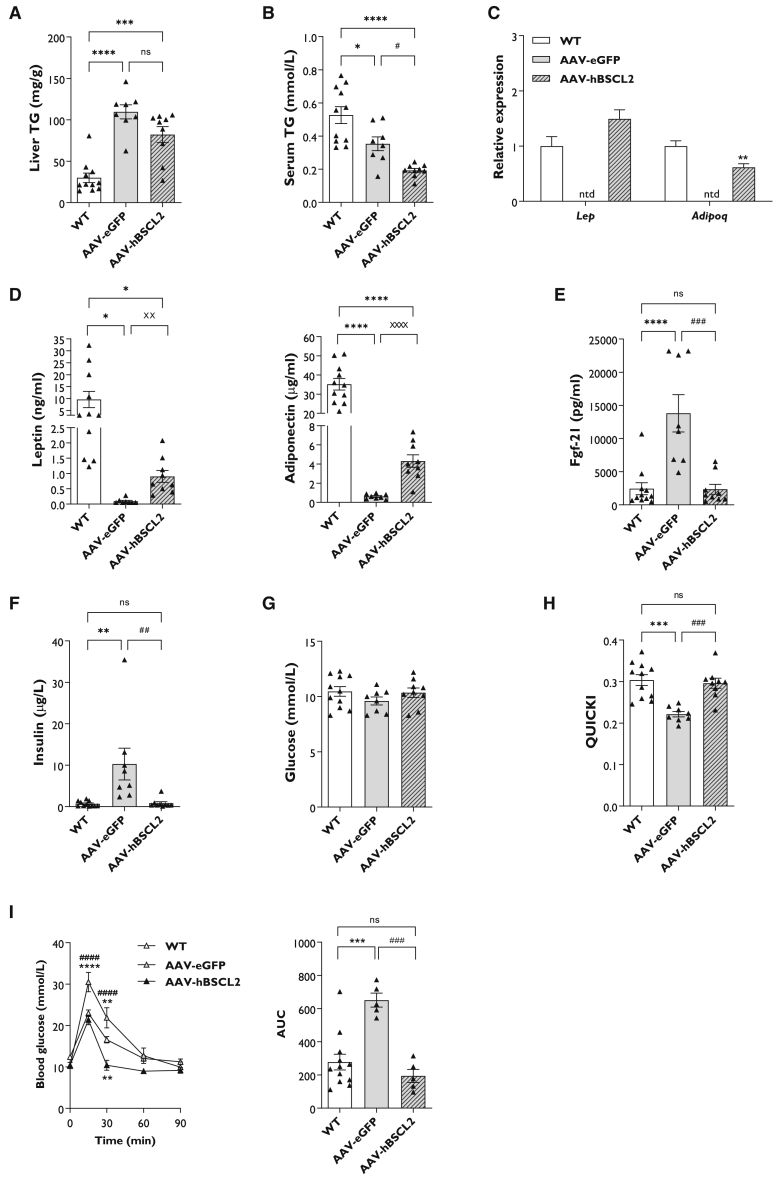
SKO mice administered gene therapy are insulin sensitive (A and B) Liver (A) and serum (B) triglyceride (TG) levels measured in WT, AAV-EGFP, and AAV-hBSCL2 mice 20 weeks after AAV administration. (C and D) Relative gene expression (C) and circulating serum levels (D) of leptin and adiponectin in WT, AAV-EGFP, and AAV-hBSCL2 mice fasted for 5 h. (E–H) Serum Fgf-21 (E), insulin (F), glucose (G), and quantitative insulin sensitivity check index (QUICKI) analysis (H) in WT, AAV-EGFP, and AAV-hBSCL2 mice fasted for 5 h. (I) Oral glucose tolerance tests and area under the curve (AUC) analysis in WT, AAV-EGFP, and AAV-hBSCL2 mice. The glucose bolus (2 g/kg) given by gavage was normalized to body weight, and AUC was calculated using the area of positive peaks of individual mice with 0-min glucose levels set as the baseline. All data are biological replicates presented as the mean ± SEM, n = 11 (WT), 8 (AAV-EGFP), and 9 (AAV-hBSCL2) mice per group for (A)–(H), n = 12 (WT), 5 (AAV-EGFP) and 5 (AAV-hBSCL2) for (I), ∗p < 0.05, ∗∗p < 0.01, ∗∗∗p < 0.001, and ∗∗∗∗p < 0.0001 versus WT, #p < 0.05, ##p < 0.01, ###p < 0.001, and ####p < 0.0001 versus AAV-EGFP, and ××p < 0.01 and ××××p < 0.0001 versus AAV-EGFP by unpaired Student’s t test. ntd, no tissue dissected.

To determine if gene therapy had any additional beneficial effects, we examined other metabolic complications known to be present in patients with BSCL2 deficiency. Despite reduced liver weights in AAV-hBSCL2 mice (Figure 3A), hepatic triglyceride (TG) content remained significantly elevated in both AAV-EGFP and AAV-hBSCL2 mice compared with WT controls. While lipid levels appeared lower in AAV-hBSCL2 mice, this was not significantly different (Figure 4A). Serum TG levels were significantly decreased in fasted AAV-EGFP mice, a previously reported observation.^8^, ^9^, ^10^ However, AAV-hBSCL2 injection significantly decreased serum TG levels further (Figure 4B).

We next examined alterations to key adipokines such as leptin and adiponectin. In gWAT, leptin expression was restored to similar levels in WT mice. Adiponectin expression was also readily detectable in AAV-hBSCL2 mice, although it was significantly lower than WT controls (Figure 4C). As expected, circulating serum leptin and adiponectin levels were significantly decreased in AAV-EGFP mice compared with WT controls. While circulating leptin and adiponectin levels were partially restored in AAV-hBSCL2 mice, they remained significantly lower than in WT mice. Nonetheless, when AAV-EGFP and AAV-hBSCL2 mice were compared directly, both adipokines were significantly increased in response to AAV-hBSCL2 injection (Figure 4D). Similar to previous reports,^10^ AAV-EGFP mice had significantly elevated levels of Fgf-21 compared with WT mice. This was completely normalized in AAV-hBSCL2 mice (Figure 4E), consistent with liver mRNA transcript alterations (Figure 3C).

Finally, we examined whether gene therapy could ameliorate insulin resistance in SKO mice. Examination of fasted circulating insulin levels revealed significant elevations in AAV-EGFP mice compared with WT controls. AAV-hBSCL2 injection restored fasting insulin levels to those observed in WT controls (Figure 4F). Fasting glucose levels were not significantly different between groups (Figure 4G). When we performed quantitative insulin-sensitivity check index (QUICKI) analysis, this was significantly reduced in AAV-EGFP mice compared with WT mice, indicative of insulin resistance. AAV-hBSCL2 treatment completely reversed insulin resistance in SKO mice (Figure 4H). Similar findings were also apparent when homeostatic model assessment of insulin resistance (HOMA-IR) analysis was performed (Figure S2G). To confirm that gene therapy improved glucose homeostasis in SKO mice, we performed an oral glucose tolerance test in a separate cohort of male and female mice 5 weeks after AAV administration. As expected, SKO mice injected with AAV-EGFP were significantly glucose intolerant compared with WT mice (Figure 4I). Gene therapy completely restored glucose tolerance in AAV-hBSCL2 SKO mice compared with AAV-EGFP mice and was even significantly improved compared with WT mice 30 min after receiving a bolus of glucose (Figure 4I).

Overall, our findings reveal that AAV-mediated gene therapy can rescue multiple metabolic complications that arise in CGL2. Importantly, a single AAV-hBSCL2 injection could restore visceral WAT development, improve hyperglycaemia, decrease serum TG levels, and promote insulin sensitivity/glucose tolerance in our pre-clinical SKO mouse model of congenital lipodystrophy.

## Discussion

Disruption of the *BSCL2* gene causes CGL2, characterized by a near-complete absence of adipose tissues. This causes severe metabolic complications, including lipoatrophic diabetes, hepatic steatosis, and cardiovascular disease.^3^^,^^5^^,^^7^^,^^23^ Numerous forms of lipodystrophy exist, and while rare, the prevalence of these conditions is likely to be substantially higher than previously thought.^24^ Few treatment opportunities currently exist, all of which only partially manage the symptoms of this devastating condition. Here, we reveal that gene therapy has the potential to provide a long-lasting and effective treatment for individuals suffering from lipodystrophy.

Using the SKO pre-clinical mouse model of CGL2, we demonstrate that a single injection with AAV bearing human *BSCL2* reversed hyperglycemia, hepatomegaly, and IR. Numerous studies have shown AAV vectors can effectively target adipose tissue depots,^18^ principally to examine gene therapies to alleviate metabolic disease in obesity.^15^, ^16^, ^17^^,^^25^ However, as WAT is essentially absent in generalized lipodystrophy, it was not clear if this approach would be effective in treating metabolic disease in this condition.^5^^,^^8^^,^^10^^,^^23^ Our findings reveal that AAV-mediated gene therapy can restore functional adipose tissue development in a pre-clinical mouse model of lipodystrophy.

The lack of gWAT in adult SKO mice strongly implies that adipose restoration by gene therapy occurs due to targeting of adipocyte progenitor cells. Consistent with this, we show that AAV8 vectors can effectively target adipose progenitor cells *in vivo*, which can differentiate into adipocytes when cultured *ex vivo*. Together, our data strongly imply that systemic delivery of human *BSCL2* using AAV vectors targets adipocyte progenitor cells, allowing adipogenesis to proceed and/or the maintenance of maturing adipocytes, which would otherwise fail due to *Bscl2* deficiency.^19^^,^^20^ Partial restoration of adiponectin and leptin levels suggests that the resultant new adipose tissue is functional. Although modest in mass, these newly developed visceral WAT depots appear sufficient to reverse metabolic complications observed in CGL2. This is consistent with previous studies showing that recovery of adipose mass by preadipocyte or adipose tissue transplantation can significantly improve metabolic health in mice lacking adipose tissue.^13^^,^^26^, ^27^, ^28^ Effective targeting of adipocyte progenitor cells also highlights the potential for repeated gene therapy administration to further increase functional adipose tissue capacity. As human adipocytes have been shown to have an average lifespan of around 10 years,^29^ restoration of adipose tissue development in patients with lipodystrophy could provide long-lasting beneficial effects to metabolic health.

While visceral adipose tissue depots were substantially restored in SKO mice receiving gene therapy, sWAT and BAT were not. It is currently unclear why this is the case, as AAV vectors can target these depots *in vivo*. Both our studies and those of others^18^ reveal that AAV serotype, route of delivery, promoter selection, and dosage of viral genome copies administered can all affect tissue targeting and the efficacy of gene expression. Future studies will be required to determine whether alternative AAV serotypes and/or tissue-specific promoters can produce more robust and diverse adipose tissue development *in vivo*. Of particular interest would be the use of novel engineered hybrid capsid serotype recombinant AAV vectors such as Rec2.^30^ Studies from Lei Cao’s laboratory have assessed the efficacy of Rec2 in adipose tissues, which revealed higher transduction efficiencies than AAV8 vectors and used systemic doses that were two orders of magnitude lower than we have used here in our studies.^16^^,^^31^

The adipose tissue microenvironment is likely to be an additional critical factor with regard to the ability of adipose tissue depots to undergo expansion. Studies performed by Jefferey and colleagues have shown that the adipose tissue microenvironment is capable of affecting adipocyte precursor activation and depot-specific adipogenesis.^32^^,^^33^ Adipose tissue depot expansion can therefore vary depending on factors such as gender and nutritional status of diets provided. It would be counterintuitive to promote adipogenesis in conditions of lipodystrophy using high-fat diets. However, combining gene therapy with PPAR gamma agonist(s) could provide a means to further enhance adipogenesis, aid the development of additional adipose tissue depots, and improve metabolic health.^10^

Despite only achieving partial adipose tissue depot development when compared with WT controls, this was sufficient to normalize hyperglycemia, decrease serum TGs, reduce hepatomegaly, and restore insulin sensitivity. These findings are consistent with our previous studies examining adipose-tissue-specific ablation of *Bscl2*.^11^^,^^34^ These mice were lipodystrophic but failed to develop severe the metabolic dysfunction observed in SKO mice. Metabolic disease also failed to manifest in adipose-tissue-specific *Bscl2* KO mice when hepatic *Bscl2* was additionally ablated using AAV to deliver Cre recombinase to the liver.^35^ We speculated that this was likely due to a failure of sufficient adipose tissue loss to cause metabolic disease. Our current findings therefore confirm that restoration of only relatively small quantities of adipose tissue may be sufficient to improve metabolic health in patients suffering from CGL2.

Leptin replacement therapy is currently the gold standard therapeutic treatment for generalized lipodystrophy.^36^^,^^37^ However, this is not widely available, and complications from treatment can develop.^38^ Upon first examination, circulating leptin levels in AAV-hBSCL2-treated mice do not appear dramatically restored when compared with WT controls of the same age. However, it is worth noting that these values are greater than leptin levels seen in WT mice at 16 weeks of age.^11^ Therefore, we believe that gene therapy leads to physiologically relevant circulating leptin levels, which are very likely capable of contributing to improved metabolic health. Adiponectin levels, on the other hand, were not restored to the levels we observed in younger WT mice. Studies have demonstrated that bone marrow adipose tissue may be the major source of circulating adiponectin.^39^ We therefore speculate that gene therapy is unlikely to have restored significant quantities of bone marrow adipose tissue, which has been shown to be severely depleted in adipose-tissue-specific *Bscl2* KO mice and patients with CGL2.^5^^,^^11^

On the other hand, we found that AAV delivery of human *BSCL2* was able to completely normalize significant elevations of circulating Fgf-21 observed in SKO mice. Recent studies have highlighted the potential therapeutic benefits of Fgf-21 administration to treat CGL2.^22^ Our data suggest that gene therapy may also be capable of promoting positive metabolic effects by reversing Fgf-21 resistance in SKO mice. While AAV vectors can effectively target the liver, previously published research indicates that hepatic expression of hBSCL2 is unlikely to be directly responsible for the improved metabolic outcomes.^21^^,^^35^ However, a recent study has suggested that AAV-mediated SEIPIN overexpression can alleviate hepatosteatosis in high-fat-diet-induced mice.^40^ Future studies will be required to determine whether beneficial effects can be derived specifically from hepatic overexpression of hBSCL2 in SKO mice.

Overall, we reveal that AAV-mediated gene therapy may have the potential to significantly improve the lives of patients suffering from lipodystrophy by partially restoring adipose development and significantly improving several aspects of metabolic disease. It will be valuable to assess other features of the condition such as infertility or chronic fatigue, which seriously impact quality of life for patients, to determine whether the beneficial effects of this treatment go beyond metabolic health. Future studies and the establishment of clinical trials, however, will be required to determine whether this could become a realistic form of medical intervention.

## Materials and methods

### Animal studies

SKO mice maintained on a C57BL/6J background were generated as previously described.^41^ Procedures on C57BL/6J and SKO mice were approved by the University of Aberdeen Ethics Review Board and performed under a project license (PPL: P1ECEB2B6) approved by the UK Home Office. Mice had *ad libitum* access to water and chow diet (CRM (P) 801722, Special Diets Services) unless otherwise stated. Mice were group housed in home cages at 20°C–24°C, 45–65% humidity, and were exposed to a 12-h/12-h light-dark period. Body composition was measured using the EchoMRI-500 analyzer (Zinsser Analytic). Fed blood glucose levels were determined by glucometer readings (AlphaTrak II, Zoetisus) from tail punctures. Tissues were rapidly dissected post-mortem, frozen in liquid nitrogen, and stored at −70°C.

### AAV administration

Ten-week-old C57BL/6J male mice were i.p. or i.v. injected at 14:00 in the home cage with AAV8-CMV-EGFP or an equivalent volume of PBS. pAAV.CMV.PI.EGFP.WPRE.bGH was a gift from James M. Wilson (Addgene viral prep #105530-AAV8). Power calculations were based on anticipated changes in glucose tolerance from values and standard deviations obtained during previous procedures. In order to attain statistical significance of p <0.01 with a power level of 80%, a sample size of eight mice per group was required. Prior to AAV administration, 10- to 14-week-old male and female SKO mice were randomized, then i.p. injected with 1 × 10^12^ genome copies of AAV8 expressing the long form of human *BSCL2* (GenBank: NM_001122955.3) under the CMV promoter (AAV8-CMV-hBSCL2, VB191203-2053snp, VectorBuilder) at 10:00 in the home cage (n = 9, 3 males, 6 females) or AAV8-CMV-eGFP (VB190926-1395dab, VectorBuilder) vector (n = 9, 4 males, 5 females), and WT littermates were used as a controls (n = 12, 7 males, 5 females).

### Oral glucose tolerance test

A cohort of 15- to 17-week-old male and female SKO mice were i.p. injected with 1 × 10^12^ genome copies of AAV-CMV-EGFP (n = 5, 3 males, 2 females) or AAV8-CMV-hBSCL2 (n = 5, 2 males, 3 females), and WT littermates were used as a controls (n = 12, 6 males, 6 females). Five weeks after AAV administration, mice were placed in clean cages, and food was withheld for 5 h. Basal glucose levels (0 min) were determined by glucometer readings (AlphaTrak II, Zoetisus) from tail punctures. Mice were then given a 2 g/kg d-glucose (Sigma-Aldrich) bolus by gavage. Blood glucose levels were monitored at 15, 30, 60, and 90 min. Mice had *ad libitum* access to water throughout.

### SVF isolation and differentiation

Ten-week-old C57BL/6J male mice were i.p. injected at 10:00 in the home cage with 1 × 10^12^ genome copies of AAV8-CMV-EGFP (VB190926-1395dab, VectorBuilder) or PBS (n = 2 mice/group). Two weeks later, mice were humanely killed by CO_2_ inhalation and cervical dislocation, sprayed with 70% ethanol, and had gonadal/subcutaneous adipose tissue excised into ice-cold PBS. All subsequent steps were performed under a laminar flow hood. Tissues were dried and minced in PBS containing 1 mg/mL collagenase D and incubated for 1 h at 37°C shaking at 200 RPM. Culture medium (DMEM high glucose supplemented with 1% Pen/Strep, 1% sodium pyruvate, 2% glutamine, and 10% FBS) was added and centrifuged at 700 × *g* for 10 min. The SVF pellet was resuspended in culture medium, filtered through a 70-μm cell strainer, centrifuged, resuspended in culture medium, and expanded at 37°C, 5% CO_2_ in a humidified incubator. Confluent SVF cells from sWAT were differentiated by supplementing culture medium with 1 μM dexamethasone, 0.5 mM 3-isobutyl-1-methylxanthine, 1 μM insulin, and 1 μM rosiglitazone. After 3 days, cells were maintained in culture medium with 1 μM insulin until day 7.

### Immunohistochemistry

Adipose tissues were fixed in 10% formalin, embedded in paraffin, and 5-μm sections cut. Sections were deparaffinized in 2 × 100% xylene, 1:1 xylene:Ethanol, 2 × 100% ethanol, 95% ethanol, 70% ethanol, 50% ethanol, 2× H_2_O for 5 min. Epitope retrieval was performed using 10 mM sodium citrate buffer (pH 6, 0.05% Tween) and incubating at 126°C for 3 min. For endogenous peroxidase quenching, sections were treated with 3% H_2_O_2_ in methanol for 15 min followed by 2× H_2_O, 2× TBS-Triton (0.025% Triton), and 2× TBS-Tween-20 (0.1% Tween-20) 5-min washes each. Frozen liver tissue sections were cut at 10 μm and fixed in 10% formalin. SVF cell cultures on coverslips were fixed in 10% formalin. After blocking in 3% BSA in TBS-T for 45 min, sections were incubated with primary antibody at 1:200–500 (anti-GFP, A-11122, Invitrogen) in 1% BSA in TBS-T at 4°C overnight. Sections were washed 3 × 5 min with TBS-T and incubated with secondary antibody at 1:500 (AF594, A-11037, Life Technologies) in 1% BSA in TBS-T for 1 h. After 2 washes with TBS-T for 5 min, DAPI (1:5000) was added, and coverslips were mounted with ProLong Diamond Antifade Mountant (Invitrogen, P36961).

### H&E staining

Deparaffinized 5-μm adipose tissue sections or 10-μm frozen liver tissue sections fixed in 10% formalin were rinsed in H_2_O and stained with hematoxylin solution (GHS316-500 mL, Sigma-Aldrich) for 1 min and washed with H_2_O for 5 min. Destaining/differentiation with 1% acid alcohol for 30 s and 1 min H_2_O, followed by blueing with saturated lithium carbonate solution for 10 s. Sections were washed with H_2_O and stained with eosin (1% eosin, 2% phloxine, 2% CuCl_2_) for 45 s. Slides were dehydrated by washing in H_2_O for 10 s, 3 × 70% ethanol, 6 × 100% ethanol, and 2 × 2 min incubation in xylene. Coverslips were mounted with CV mount (Leica, 14046430011).

### Serum analysis

Blood was collected in SST amber tubes (BD Microtainer) from 5-h-fasted mice by cardiac puncture at 13:00, inverted, and incubated at room temperature for 30 min. Samples were centrifuged at 12,000 × *g* for 10 min and serum collected. Insulin, glucose, adiponectin, leptin, and Fgf-21 analysis was performed at the Core Biochemical Assay Laboratory (Cambridge, UK). Serum TG levels were determined using the Triglyceride Liquid Assay (Sentinel Diagnostics). QUICKI was calculated as previously described.^42^ QUICKI = 1/[log(I_0_) + log(G_0_)], where I_0_ is fasting insulin (μU/mL) and G_0_ is fasting glucose (mg/dL). HOMA-IR was calculated using fasting glucose (mmol/L)∗fasting insulin (μU/mL)/22.5.

### Gene expression

RNA was extracted from frozen tissues using the RNeasy mini kit (Qiagen). Equal quantities of RNA were DNase I treated (Sigma), reverse transcribed with M-MLV reverse transcriptase, 5× reaction buffer, dNTPs, and random primers (Promega). Real-time quantitative PCR was performed on the CFX384 Touch Real-Time PCR Detection System (BioRad). No template and no reverse transcriptase controls were performed for every gene analyzed. The geometric mean of three stable reference genes (*Nono*, *Ywhaz*, and *Hprt*) was used for normalization.

### Liver TG assay

Frozen liver tissue samples were weighed, homogenized in 1 mL PBS, and kept on ice. Liver lysates were centrifuged at 12,000 × *g* for 10 min at 4°C. Supernatants were collected and TG levels determined using the Triglyceride Liquid Assay (Sentinel Diagnostics) and normalized to tissue weight.

### Western blot

Frozen tissues were homogenized in radioimmunoprecipitation assay (RIPA) buffer containing cOmplete protease inhibitor (Roche). Protein concentrations were determined by BCA assay (Thermo Fisher Scientific). SDS-PAGE was performed using equal quantities of protein transferred to a PVDF membrane. Antibodies used at a dilution of 1 in 1,000 included anti-BSCL2/seipin, which is only capable of reacting with human BSCL2/seipin (#23846, Cell Signaling), anti-GFP (A-11122, Invitrogen), and anti-calnexin (ab75801, Abcam). Anti-rabbit HRP secondary antibody was used at a dilution of 1 in 5,000 (#7074, Cell Signaling) and visualized using enhanced chemiluminescence (Immobilon Crescendo Western HPR Substrate, Millipore, #WBLUR0500).

### Statistical analyses

All data are presented as mean ± SEM and analyzed by unpaired two-tailed Student’s t test, one-way ANOVA with Tukey’s post-hoc test, or two-way repeated-measures ANOVA with Bonferroni post-hoc test as appropriate using GraphPad Prism. A p <0.05 was considered statistically significant.

## Data availability

The datasets generated and/or analyzed during the current study are available from the corresponding author on reasonable request.

## Supplemental information

Document S1. Figures S1 and S2

Document S2. Article plus supplemental information
