# Supplementary material for: Gene therapy restores adipose tissue and metabolic health in a pre-clinical mouse model of lipodystrophy
Source: Mol Ther Methods Clin Dev. 2022 Oct 3;27:206–16. doi: 10.1016/j.omtm.2022.09.014 (PMC9589143; doi:10.1016/j.omtm.2022.09.014)
Supplement: Document S1. Figures S1 and S2 [file mmc1.pdf]

OMTM, Volume *27*

Supplemental information

Gene therapy restores adipose tissue and metabolic health in a pre-clinical mouse model of lipodystrophy

Nadine Sommer, Ahlima Roumane, Weiping Han, Mirela Delibegović, Justin J. Rochford, and George D. Mcilroy

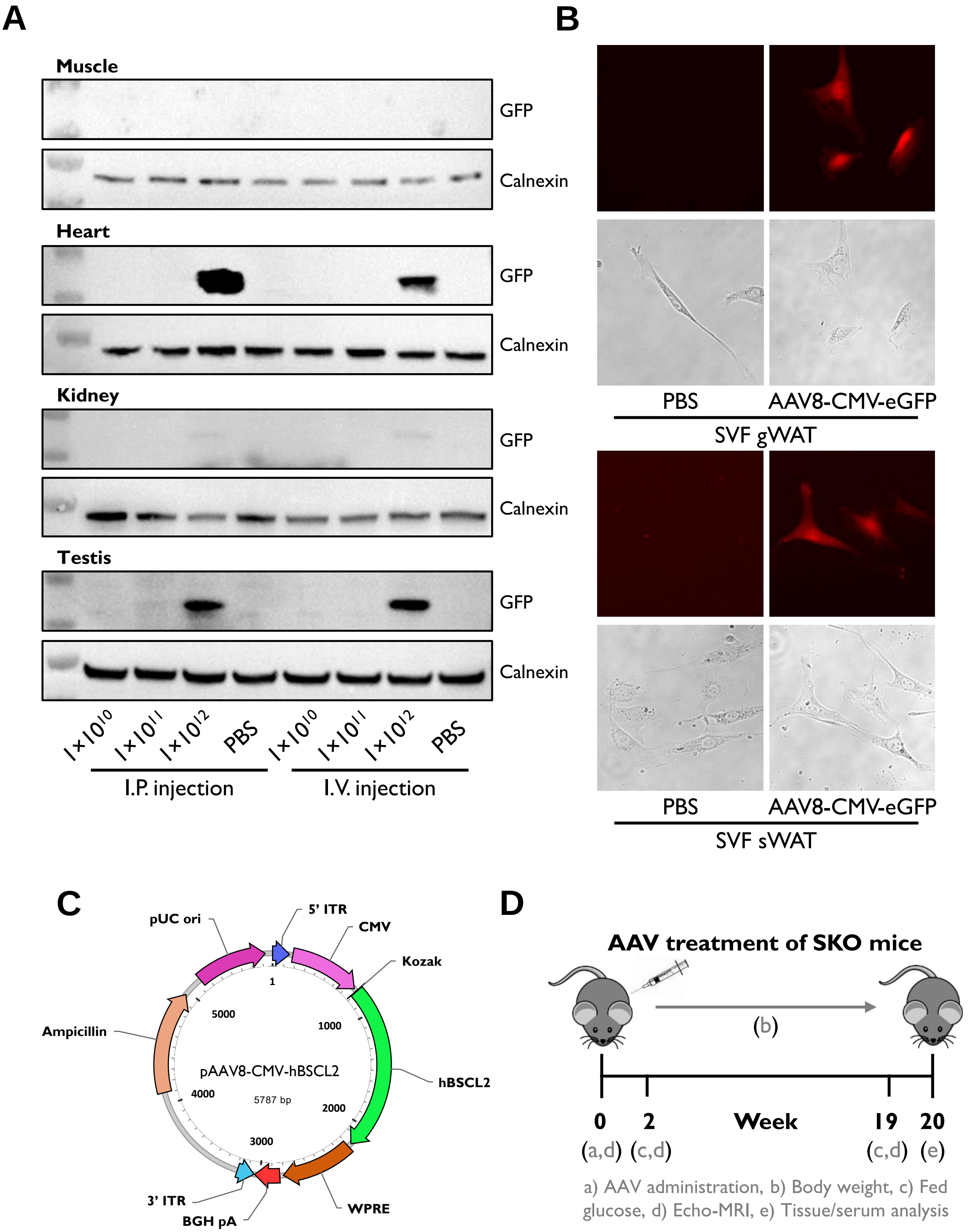
A
Muscle
GFP
Calnexin
Heart
GFP
Calnexin
Kidney
GFP
Calnexin
Testis
GFP
Calnexin
1×10^10
1×10^11
1×10^12
PBS
1×10^10
1×10^11
1×10^12
PBS
I.P. injection
I.V. injection
B
PBS
AAV8-CMV-eGFP
SVF gWAT
PBS
AAV8-CMV-eGFP
SVF sWAT
C
pUC ori
5' ITR
CMV
Kozak
Ampicillin
hBSCL2
3' ITR
BGH pA
WPRE
pAAV8-CMV-hBSCL2
5787 bp
1
1000
2000
3000
4000
5000
D
AAV treatment of SKO mice
(b)
0
2
Week
19
20
(a,d)
(c,d)
(c,d)
(e)
a) AAV administration, b) Body weight, c) Fed glucose, d) Echo-MRI, e) Tissue/serum analysis

Figure S1: Adeno-associated virus delivery of eGFP to murine tissues. (**A**) Western blot analysis of eGFP levels in muscle, heart, kidney and testis. Male mice were injected with AAV8 vectors overexpressing eGFP from the mammalian cytomegalovirus (CMV) promoter (AAV8-CMV-eGFP). Intraperitoneal (I.P.) or intravenous (I.V.) injections were given using 1×10^{10}, 1×10^{11} or 1×10^{12} genome copies of AAV, an equivalent volume of PBS was used as a control. (**B**) eGFP fluorescence and phase contrast images of live stromal vascular fraction primary cell cultures 72 hours after isolation from gWAT and sWAT of mice injected with 1×10^{12} genome copies of AAV8-CMV-eGFP or an equivalent volume of PBS by I.P. (**C**) Viral vector plasmid design to overexpress the long form of the human *BSCL2* transcript (NM_001122955.3) driven by the CMV promoter (pAAV8-CMV-hBSCL2). (**D**) Graphical representation of the experimental strategy used to examine whether gene therapy can rescue metabolic dysfunction in seipin knockout mice (SKO).

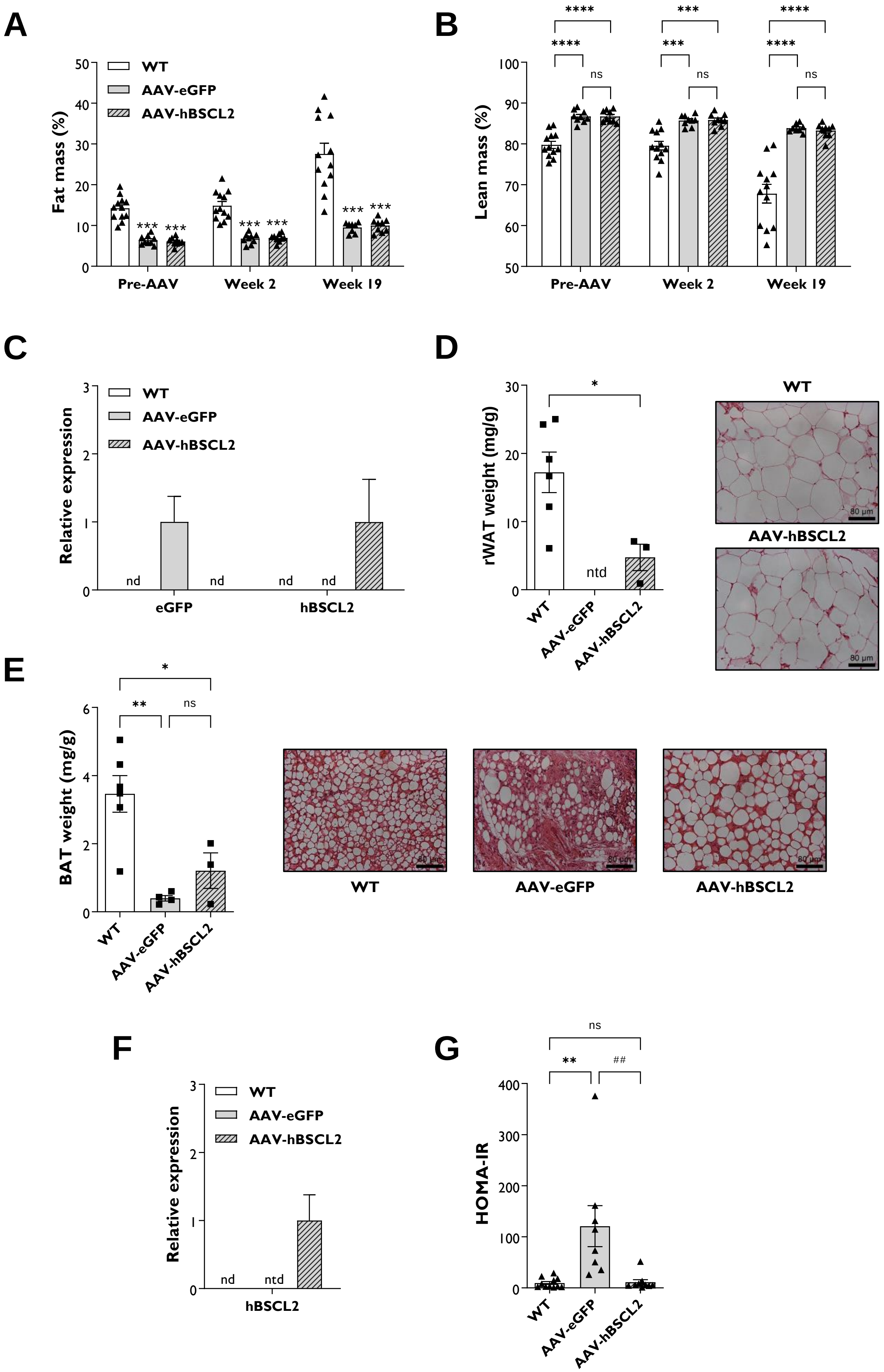
A
B
C
D
E
F
G
Fat mass (%)
WT
AAV-eGFP
AAV-hBSCL2
Pre-AAV
Week 2
Week 19
Lean mass (%)
Relative expression
eGFP
hBSCL2
nd
rWAT weight (mg/g)
ntd
BAT weight (mg/g)
HOMA-IR
ns
##

Figure S2: Characterisation of seipin knockout mice treated with gene therapy. Whole body fat mass (**A**) and whole body lean mass (**B**) levels assessed by Echo-MRI and normalised to body weight prior to gene therapy (Pre-AAV), two and nineteen weeks after AAV administration in WT, AAV-eGFP and AAV-hBSCL2 mice. Relative gene expression levels of eGFP and hBSCL2 in the liver (**C**). Tissue weight and H&E sections of (**D**) retroperitoneal white adipose tissue (rWAT) and (**E**) brown adipose tissue (BAT) from WT, AAV-eGFP and AAV-hBSCL2 male mice twenty weeks after AAV administration. Scale bar represents 80 µm. Relative gene expression levels of hBSCL2 in gWAT (**F**). Homeostatic model assessment of insulin resistance (HOMA-IR) analysis of WT, AAV-eGFP and AAV-hBSCL2 mice twenty weeks after AAV administration (**G**). All data are biological replicates presented as the mean ± SEM, n = 11-12 (WT), 8-9 (AAV-eGFP) and 9 (AAV-hBSCL2) mice per group for A, B, C, F and G, n = 6 (WT), 4 (AAV-eGFP) and 3 (AAV-hBSCL2) per group for D and E, *p<0.05, ** p<0.01, *** p<0.001 and **** p<0.0001 vs WT, ## p<0.01 vs AAV-eGFP, ntd = no tissue dissected, nd = not detected.
